# Symbiotic Performances of Three *Mesorhizobium huakuii* Strains Inoculated to Chinese Milk Vetch Varieties

**DOI:** 10.3389/fpls.2020.599400

**Published:** 2020-12-17

**Authors:** Fang Liu, Mingxuan Yi, Xinbao Liu, Yixin Shen, Jianlong Li, Hui Wang, Dianlin Yang, Zhengguo Sun

**Affiliations:** ^1^College of Agro-Grassland Science, Nanjing Agricultural University, Nanjing, China; ^2^Sheep Industry Development Center of Yulin City, Yulin, China; ^3^School of Life Sciences, Nanjing University, Nanjing, China; ^4^Agro-Environmental Protection Institute, Ministry of Agriculture and Rural Affairs, Key Laboratory of Original Agro-Environmental Pollution Prevention and Control, Tianjin Key Laboratory of Agro-Environment and Agro-Product Safety, Tianjin, China

**Keywords:** Chinese milk vetch, rhizobium strain, exclusivity, biomass, nodule characteristics, nitrogenase activity

## Abstract

In this experiment, 4 varieties of Chinese milk vetch (Xinyang, Minzi No. 6, Minzi 8487711, and Shishou) were used as host plants and inoculated with 3 strains of rhizobium (CCBAU 2609, M. h 93, and 7653R). The differences in their morphology, yield, and nodule characteristics at different growth stages were studied and the reasons for these differences were analyzed to explore the exclusivity between different varieties of Chinese milk vetch and strains of rhizobium. Results showed no significant difference in plant height and whole-plant dry weight at seedling stage under different treatments but significant differences in these characteristics at full flowering stage. The results indicated that Minzi 8487711 and Shishou were significantly better than the other varieties. During the whole growth period, the growth indexes and nodule characteristics of the 4 varieties inoculated with strain 7653R were better than those of the varieties inoculated with other strains. At full flowering stage, compared with that of the control group, the biomasses of Xinyang, Minzi No.6, Minzi 8487711, and Shishou had increased by 2.04, 2.84, 1.56, and 2.69-fold, respectively, and nitrogenase activities increased by 3.82, 9.60, 6.21, and 15.18%, respectively. Significant differences in the exclusivity between varieties and strains were observed. Minzi No.6–7653R and Shishou variety–7653R had the strongest exclusivity. The results showed that 7653R was a broad-spectrum and high-efficiency rhizobium strain. 7653R is recommended to be used in combination with Chinese milk vetch varieties, such as Minzi 8487711 and Shishou, to obtain advantages in yield and nitrogen fixation in production.

## Introduction

Chinese milk vetch (*Astragalus sinicus* L.), a green manure plant with early florescence, colorful flowers, and high biomass production, is planted in autumn and winter (Shim and Kang, 2004; Kim et al., 2007; Lee et al., 2015). It is connected closely with rice stubble in the middle and lower reaches of the Yangtze River. In the Chinese milk vetch–rice rotation model, milk vetch can improve soil fertility, improve soil physical and chemical properties, and promote the yield stability and quality improvement of the succeeding rice crop. Chinese milk vetch is often called “paddy field mate” (Piotrowska and Wilczewski, 2012; Xie et al., 2016). In recent years, the cultivated land area of Chinese milk vetch in the south area of the Yangtze River has increasingly expanded with the promotion of planting structure adjustment, crop rotation, and land fallowing by the government. At the end of 2018, 5 cities in southern Jiangsu Province in the lower reaches of the Yangtze River took integrated measures to promote crop rotation and land fallowing. Since then, Chinese milk vetch and other green manure plants have covered an area of approximately 30,000 hectares. However, according to field investigations, Chinese milk vetch in many areas of southern Jiangsu Province generally has low grass yield, weak stress resistance, and limited nodule growth and nitrogen fixation. These problems may be attributed to 2 reasons. First, this region suffers from extremely cold and rainy winter. Second, rhizobium strains with exclusivity have not been applied for a long term.

In contrast to plants in Gramineae and other families and genera, Chinese milk vetch, a leguminous plant, possesses a special, stable, and important symbiotic nitrogen fixation system that involves its roots and nodules (Yoshioka and Maruyama, 1990; Brett et al., 2010; Zhu et al., 2014). Rhizobia participate in biological nitrogen fixation and the carbon and nitrogen cycles by infecting the roots of Chinese milk vetch through creating nodules, thus converting free nitrogen in the air into ammonium nitrogen that can be directly absorbed and utilized by roots for plant growth (Brewin, 1991; Haag et al., 2013; Smith et al., 2018). The United States, Japan, Australia, and other developed countries have reached a consensus that leguminous plants should be inoculated with their corresponding exogenous rhizobium strain with a certain exclusivity when planted. This approach can promote the accumulation of photosynthetic products, obviously improve nitrogen fixation efficiency, and effectively increase biological yield. It is a highly effective, environmentally friendly, and safe technical measure with prospects for development and application (Nakayama and Yoshida, 2005; Kim et al., 2007; Asagi and Ueno, 2009). However, rhizobium application, which can increase yield and efficiency, is usually ignored. Incomplete statistics show that the proportion of leguminous plants inoculated with commercial rhizobia in China remains less than 3%. Although a consensus has been reached earlier on the importance and necessity of studies on the exclusivity of Chinese milk vetch varieties and strains, their achievements are few, and some relevant practical problems existing in production still cannot be solved. This limitation may be caused by planting scale, growth environment, variety, strain, and other factors. First, compared with that of other crops, the area of Chinese milk vetch in the middle and lower reaches of the Yangtze River is often smaller. The planting area of Chinese milk vetch has increased to a certain extent in recent years after its sharp compression in the 1970s and 1980s. Second, most existing rhizobium strains and Chinese milk vetch varieties are tested and screened in orchards, whereas few varieties and matching strains have been tested in sticky rice stubble soil. Third, matching rhizobium strains have failed to keep pace with the updating of Chinese milk vetch varieties. Previous studies have confirmed that the growth and nitrogen fixation effects of the same rhizobium strain, when inoculated into different varieties, may be different (Kulkarni and Nautiyal, 2000; Olsthoorn et al., 2000). Fourth, given their low nitrogen fixation efficiency, existing rhizobium strains and their related products are not competitive with indigenous rhizobium. Related studies have shown that 75% of indigenous rhizobium strains have low effectiveness or are even ineffective in matching with existing Chinese milk vetch varieties. Therefore, we must analyze the internal and external causes of these problems. We must effectively promote research on the variety renewal of Chinese milk vetch, the extraction and identification of high-efficiency rhizobium strains, and exclusivity between varieties and strains. High-efficiency variety–strain combinations should be screened out.

Against this background, in this study, four representative varieties of Chinese milk vetch were selected and then correspondingly inoculated with 3 excellent rhizobium strains to measure and analyze their plant growth effect and nodule characteristics at different growth stages. This research can provide reliable reference for the scientific introduction and efficient planting of Chinese milk vetch in southern Jiangsu and other areas in the middle and lower reaches of the Yangtze River.

## Materials and Methods

### Materials Tested

Experiments were conducted by using 4 representative varieties of Chinese milk vetch that are planted in the middle and lower reaches of the Yangtze River, specifically southern Jiangsu, in recent years. These varieties have a certain planting area, early florescence, and large leaf mass. Details are given in Table 1. In this study, three rhizobium strains, coded as CCBAU 2609, M. h 93, and 7653R, from Microbiology Laboratory, Nanjing Agricultural University, were evaluated. Details are given in Table 2. The culture medium used for growing rhizobium was Yeast Mannitol Agar (YMA) medium, and bacterial liquid was cultured by YMA liquid medium.

**TABLE 1:** Four Chinese milk vetch cultivars used in this study.

Cultivars	Material type	Growth period (day)	Sources of material	Coordinate position
Xinyang variety	Local variety	158	Xinyang City Academy of Agricultural Sciences	E 114°4′47″, N 32°6′21″
Minzi No.6	Cultivated variety	163	Soil and Fertilizer Institute of Fujian Academy of Agricultural Sciences	E 119°18′48″, N 20°6′34″
Minzi 8487711	Cultivated strain	167	Soil and Fertilizer Institute of Fujian Academy of Agricultural Sciences	E 119°18′48″, N 20°6′34″
Shishou variety	Local variety	160	Collected in Farmland of Shishou City, Jingzhou City, Hubei Province	E 112°31′59″, N 29°43′32″

**TABLE 2:** Origin of the three mesorhizobial strains used in this study.

Rhizobium strain	Scientific name	Host species	Source
CCBAU 2609	*Mesorhizobium huakui* CCBAU 2609 (Jia et al., 2015)	*Astragalus sinicus* L.	The lab of Department of Microbiology
M. h 93	*Mesorhizobium huakui 93* (Ji et al., 2010)	*Astragalus sinicus* L.	The lab of Department of Microbiology
7653R	*Mesorhizobium huakui* 7653R (Wang et al., 2014)	*Astragalus sinicus* L.	The lab of Department of Microbiology

### Methods

The pot experiment was conducted in Pailou Teaching and Research Base, Nanjing Agricultural University, on November 1, 2018. The pots used in this experiment were 30 cm in diameter and 30 cm in height. The experiment was conducted with 4 Chinese milk vetch varieties and 4 rhizobium treatments (including CK treatments without inoculation), with a total of 16 treatments. Considering the need for multiple samples in different growth stages, 9 pots were allocated to each treatment, and a total of 144 pots were planted. Soil was sieved, evenly mixed with river sand at 3:2 ratio, filled into pots for watering, and then balanced for 2 days. Chinese milk vetch seeds were sterilized with 5% sodium hypochlorite for 8 min and washed with sterile distilled water. Ten seeds were sown per pot, covered with a 1 cm sampled soil. After the expansion of the first true leaf, 6 final seedlings were arranged in 2 rows with the same interval.

Rhizobia were rejuvenated on YMA slants; transferred to YMA liquid medium; and subjected to shaking incubation at 28°C and 220 rpm for 2–4 days until the culture OD_600_ reached 0.8–1.0. When the first leaf was fully expanded, 5 mL of rhizobium solution was inoculated into the roots with sterile water as the control.

### Content Determination and Methods

Whole plants were sampled at the seedling stage, branching stage, and full flowering stage. After rinsing, the roots were transported back to the laboratory in an ice box. Plant height, fresh weight above ground, whole-plant dry weight, nodule weight, nodule number, and nitrogenase activity were measured.

Nitrogenase activity was determined via the acetylene reduction method. A root nodule with a weight of approximately 0.2 g was transferred into a 10 mL sealed bottle with good air tightness. Subsequently, 1 mL of air was absorbed by using a sealed needle tube, and 1 mL of high-purity acetylene gas (10%) was added at the same time. Then the bottle was sealed, inverted, and transferred into an incubator at 28°C for 2 h. Then, for detection, 1 mL of the reaction gas was removed and placed in a 10 mL head space bottle from which 1 mL of air had been removed. In the treatment group, 100 μL gas samples were pipetted to determine the peak value of ethylene and acetylene by using a HP 6890 Series Gas Chromatograph System (Chromatographic column: Anyin, PLOTAL2O3, 30 m × 0.53 mm × 25 μm).

The operating conditions were as follows: detection room temperature of 100°C, column temperature of 100°C, gas flow rate of 400 mL⋅min^–1^, N_2_ flow rate of 50 mL⋅min^–1^, and H_2_ flow rate of 30 mL⋅min^–1^. All samples were dried and weighed to calculate nitrogenase activity.

Nitrogenase activity was determined as follows:

$$C_{2}\,H_{4}\cdot \text{nmol}=\frac{K\,\left(T+X\right)}{T}\times \frac{y}{760}\times \frac{z}{\left(2.24\times 10^{-6}\right)\,W}\times \frac{1}{t}$$

where z is the injected acetylene milliliters (mL); W is the test sample dry weight (g); t is the reaction time after the injection of acetylene (min); 22.4 represents the volume of 1 g of molecular gas at absolute temperature and 1 atmospheric pressure (L⋅mol^–1^); K is the ratio of the ethylene peak value to the acetylene peak value; T is the absolute temperature; X is the actual temperature at the time of determination (°C); y represents the atmospheric pressure at the time of determination (Pa); and 760 is a constant, representing standard atmospheric pressure.

### Data Processing

Data were processed by using Microsoft Office Excel (Excel 2010) and SPSS analytical software (SPSS 20.0), and figures were generated by Origin (Origin pro 2017). The results were expressed as mean ± standard error (SE). One-way ANOVA with Duncan’s test at *P* < 0.05 was then used to compare the differences in means among treatments.

## Results

### Different Responses of the Morphology, Yield, and Nodule Characteristics of Different Varieties of Chinese Milk Vetch at the Seedling Stage to Inoculation With Different Rhizobia

As shown in Table 3, plant height, aboveground fresh weight, whole-plant dry weight, and nodule number and weight per plant at the seedling stage were observed after the inoculation of the 4 varieties of Chinese milk vetch with 3 strains of rhizobium.

**TABLE 3:** Growth indexes, biomass, and root nodule characteristics at the seedling stage of Chinese milk vetch cultivars inoculated with different *Mesorhizobium huakuii* strains.

Cultivars	Strains	Plant height (cm)	Fresh weight above aground (g⋅plant^–1^)	Whole-plant dry weight (g⋅plant^–1^)	Nodule number (No. plant^–1^)	Nodule weight (mg⋅plant^–1^)
Xinyang variety	CCBAU2609	7.47 ± 0.37^ab^	1.13 ± 0.13^ab^	0.32 ± 0.04^a^	18.00 ± 10.39^b^	36.11 ± 7.22^b^
	M. h93	7.33 ± 0.28^b^	0.89 ± 0.08^b^	0.28 ± 0.03^a^	7.89 ± 3.91^b^	10.00 ± 5.09^bc^
	7653R	8.79 ± 0.15^a^	1.56 ± 0.21^a^	0.43 ± 0.06^a^	88.50 ± 10.87^a^	81.11 ± 15.56^a^
	CK	8.38 ± 0.67^ab^	1.42 ± 0.20^ab^	0.37 ± 0.06^a^	0.06 ± 0.02^b^	4.44 ± 0.32^c^
Minzi No.6	CCBAU2609	8.50 ± 0.53^a^	1.76 ± 0.30^a^	0.52 ± 0.07^a^	0.11 ± 0.06^b^	1.67 ± 0.96^b^
	M. h93	8.10 ± 0.34^a^	1.31 ± 0.01^a^	0.39 ± 0.01^a^	0.22 ± 0.15^b^	2.78 ± 1.47^b^
	7653R	8.62 ± 0.25^a^	1.39 ± 0.15^a^	0.38 ± 0.03^a^	100.50 ± 4.78^a^	60.56 ± 2.00^a^
	CK	8.49 ± 0.49^a^	1.85 ± 0.22^a^	0.48 ± 0.05^a^	0.28 ± 0.15^b^	1.11 ± 0.07^b^
Minzi 8487711	CCBAU2609	9.23 ± 1.39^a^	1.52 ± 0.34^a^	0.40 ± 0.07^a^	1.33 ± 0.69^b^	18.33 ± 8.39^b^
	M. h93	9.38 ± 0.45^a^	1.68 ± 0.09^a^	0.39 ± 0.04^a^	56.22 ± 3.51^b^	127.22 ± 14.77^ab^
	7653R	11.23 ± 0.20^a^	1.46 ± 0.13^a^	0.33 ± 0.03^a^	199.94 ± 5.15^a^	241.67 ± 35.28^a^
	CK	9.71 ± 0.30^a^	2.24 ± 0.35^a^	0.45 ± 0.07^a^	4.78 ± 3.46^b^	3.33 ± 1.67^b^
Shishou variety	CCBAU2609	10.34 ± 0.14^b^	2.48 ± 0.28^a^	0.55 ± 0.07^a^	1.72 ± 0.06^b^	23.89 ± 0.32^b^
	M. h93	9.00 ± 0.26^c^	2.22 ± 0.17^a^	0.46 ± 0.06^ab^	5.61 ± 0.30^b^	4.44 ± 0.14^b^
	7653R	11.59 ± 0.39^a^	2.25 ± 0.11^a^	0.52 ± 0.04^ab^	172.50 ± 18.56^a^	72.78 ± 6.76^a^
	CK	10.91 ± 0.52^ab^	1.52 ± 0.19^b^	0.35 ± 0.02^b^	0.01 ± 0.00^b^	0.01 ± 0.00^b^

**Data are means ± standard error around the mean. The columns were tested separately. The same letters indicate the lack of significant difference (P > 0.05), whereas different letters denote significant difference between corresponding values (P < 0.05)*.*

After inoculation with CCBAU 2609 and M. h 93, the Xinyang variety only showed a significant increase in the weight of nodules per plant. The number of nodules per plant also increased significantly after inoculation with 7653R.

No significant changes were found in all indexes of Minzi No. 6 and Minzi 8487711 after inoculation with CCBAU 2609 and M. h 93. However, the number and weight of nodules per plant of these varieties increased significantly after inoculation with 7653R.

After inoculation with CCBAU 2609, the aboveground fresh weight and the dry weight of whole plants of the Shishou variety were significantly higher than those of CK plants. Plant height and aboveground fresh weight significantly increased after inoculation with M. h 93. Aboveground fresh weight and nodule number and weight per plant significantly increased after inoculation with 7653R.

### Differential Responses of the Morphology, Yield, and Nodule Characteristics of Varieties at the Branching Stage to Inoculation With Strains of Rhizobium

The plant height, grass yield, and nodule characteristics of different varieties of Chinese milk vetch at the branching stage changed after inoculation with different rhizobia (Table 4). After inoculation with 7653R, the plant height, aboveground fresh weight, whole plant dry weight, and nodule number and weight per plant of the 4 varieties were significantly higher than those of their respective CK plants. Except for the aboveground fresh weight index, the indexes of Xinyang variety, after inoculation with CCBAU 2609, did not change significantly, and the above-mentioned indexes did not increase significantly after inoculation with M. h 93. The plant height and aboveground fresh weight of Minzi NO.6 inoculated with CCBAU 2609 were significantly higher than those of CK plants. However, the whole plant dry weight and nodule number and weight per plant of Minzi No.6 inoculated with CCBAU 2609 did not increase significantly. After inoculation with M. h 93, plant height, aboveground fresh weight, and whole plant dry weight increased significantly, and nodule number and weight per plant did not increase significantly. No significant difference between the 2 rhizobium strains was observed, and the trend was similar. The aboveground fresh weight and whole plant dry weight of Minzi 8487711 increased significantly after inoculation with CCBAU 2609, and all indexes increased significantly after inoculation with M. h 93. However, the increase after inoculation with 7653R was larger than that after inoculation with other strains. A consistent change trend was observed in Shishou inoculated with CCBAU 2609 and M. h 93, and only some indexes, such as aboveground fresh weight and nodule weight per plant, of this plant were significantly higher than those of CK plants.

**TABLE 4:** Growth indexes, biomass, and root nodule characteristics at the branching stage of Chinese milk vetch cultivars inoculated with different *Mesorhizobium huakuii* strains.

Cultivars	Strains	Plant height (cm)	Fresh weight above aground (g⋅plant^–1^)	Whole-plant dry weight (g⋅plant^–1^)	Nodule number (No. plant^–1^)	Nodule weight (mg⋅plant^–1^)
Xinyang variety	CCBAU2609	20.24 ± 0.31^b^	4.76 ± 0.07^b^	0.85 ± 0.05^ab^	66.44 ± 38.01^b^	129.44 ± 72.74^b^
	M. h93	18.44 ± 0.73^b^	4.10 ± 0.24^bc^	0.87 ± 0.02^ab^	2.06 ± 0.18^b^	75.00 ± 2.76^b^
	7653R	24.75 ± 1.44^a^	7.16 ± 1.18^a^	1.15 ± 0.18^a^	288.08 ± 25.78^a^	587.78 ± 10.02^a^
	CK	17.15 ± 1.23^b^	2.62 ± 0.30^c^	0.70 ± 0.09^b^	0.01 ± 0.00^b^	13.89 ± 2.40^b^
Minzi No.6	CCBAU2609	16.91 ± 0.37^b^	4.29 ± 0.06^b^	0.78 ± 0.05^bc^	4.33 ± 0.76^b^	118.89 ± 68.19^b^
	M. h93	17.06 ± 1.01^b^	4.91 ± 0.66^b^	0.91 ± 0.07^ab^	45.94 ± 30.60^b^	402.78 ± 77.82^b^
	7653R	20.53 ± 0.74^a^	6.27 ± 0.46^a^	1.04 ± 0.11^a^	149.78 ± 30.60^a^	546.11 ± 29.00^a^
	CK	13.83 ± 0.62^c^	2.72 ± 0.19^c^	0.59 ± 0.04^c^	1.72 ± 0.24^b^	0.56 ± 0.08^b^
Minzi 8487711	CCBAU2609	25.87 ± 1.72^b^	5.13 ± 0.25^b^	0.99 ± 0.04^c^	25.50 ± 2.50^c^	150.00 ± 38.24^b^
	M. h93	30.30 ± 1.00^a^	5.78 ± 0.01^b^	1.20 ± 0.03^b^	179.72 ± 75.01^b^	88.89 ± 65.67^a^
	7653R	32.39 ± 0.49^a^	6.96 ± 0.55^a^	1.40 ± 0.02^a^	356.76 ± 48.92^a^	183.33 ± 40.73^a^
	CK	23.43 ± 1.63^b^	3.49 ± 014^c^	0.79 ± 0.04^d^	0.13 ± 0.03^c^	95.00 ± 42.21^b^
Shishou variety	CCBAU2609	24.04 ± 1.64^b^	5.26 ± 0.69^b^	1.21 ± 0.15^b^	1.78 ± 0.22^b^	114.44 ± 31.11^b^
	M. h93	26.22 ± 1.96^b^	6.59 ± 1.07^b^	1.29 ± 0.32^b^	43.61 ± 0.53^b^	116.67 ± 4.41^b^
	7653R	32.28 ± 0.61^a^	10.51 ± 0.98^a^	2.20 ± 0.18^a^	341.39 ± 52.64^a^	519.44 ± 27.99^a^
	CK	20.97 ± 2.37^b^	2.38 ± 0.61^c^	0.69 ± 0.14^b^	0.39 ± 0.15^b^	32.78 ± 18.77^c^

**Data are means ± standard error around the mean. The columns were tested separately. The same letters indicate the lack of significant difference (P > 0.05), whereas different letters denote significant difference between corresponding values (P < 0.05).**

### Differential Responses of the Morphology, Yield, and Nodule Characteristics of Varieties at Flowering Stage to Inoculation With Strains of Rhizobium

Some differences were observed in morphology, yield, and nodule characteristics at flowering stage among different varieties of Chinese milk vetch after inoculation with different strains of rhizobium (Table 5). Under the condition that Minzi 8487711 and Shishou were not inoculated with exogenous rhizobia, these varieties had certain advantages over the other 2 varieties in terms of plant height, above ground fresh weight, whole plant dry weight, and other morphology and yield indexes. The plant height, aboveground fresh weight, whole plant dry weight, and nodule indexes of Xinyang inoculated with CCBAU 2609, M. h 93, and 7653R were higher than those of the control group. The plant heights of Xinyang inoculated with CCBAU 2609, M. h 93, and 7653R increased by 35.99, 17.84, and 46.40%, respectively, compared with those of CK plants. After inoculation with CCBAU 2609, M. h 93, and 7653R, the aboveground dry weights of Xinyang increased by 96.70, 38.83, and 159.34%, respectively, and the dry weights of the whole plant increased by 45.07, 18.31, and 102.82%, respectively. After inoculation with CCBAU 2609, M. h 93, and 7653R, the number of nodules per plant increased by 280.80, 169.61, and 351.48%, respectively, and nodule weights increased by 75.25, 39.25, and 119.32% respectively, among which the indexes after inoculation with 7653R were significantly higher than those after the control treatment. No significant change in plant height, dry weight, and nodule number was observed, although above ground fresh weight and nodule weight significantly increased after inoculation with CCBAU 2609. All indexes mentioned above changed indistinctively after inoculation with M. h 93. After inoculation with 7653R, Minzi No.6 showed significant increases of 49.73, 172.26, 182.42, 7011.94, and 519.66% in plant height, aboveground fresh weight, whole plant dry weight, nodule number, and nodule weight and other indexes, respectively, compared with CK. In terms of most indexes, 7653R had an absolute advantage over the other 2 strains of rhizobium. The plant morphology, yield, and nodule indexes of Minzi 8487711 increased significantly after inoculation with 7653R and M. h 93. After inoculation with CCBAU 2609, only above ground fresh weight and whole-plant dry weight increased significantly, but plant height, nodule number, and nodule weight did not change significantly. All of the indexes of Shishou also increased significantly after inoculation with 7653R. However, only some indexes increased significantly after inoculation with CCBAU 2609 and M. h 93. The above indexes indicated that the exclusivity between 7653R and the 4 tested varieties was better than that between the other strains and the 4 tested varieties, and CCBAU 2609 had good exclusivity only with Xinyang. M. h 93 had certain exclusivity with Minzi No.6 and Minzi 8487711, but its exclusivity with the other 2 varieties was poor.

**TABLE 5:** Growth indexes, biomass, and root nodule characteristics at the full flowering stage of Chinese milk vetch cultivars inoculated with different *Mesorhizobium huakuii* strains.

Cultivars	Strains	Plant height (cm)	Fresh weight above aground (g⋅plant^–1^)	Whole-plant dry weight (g⋅plant^–1^)	Nodule number (No. plant^–1^)	Nodule weight (mg⋅plant^–1^)
Xinyang variety	CCBAU2609	39.71 ± 0.30^a^	5.37 ± 0.28^b^	1.03 ± 0.07^b^	269.11 ± 20.03^a^	574.44 ± 2.78^b^
	M. h93	34.41 ± 1.26^b^	3.79 ± 0.14^c^	0.84 ± 0.05^bc^	190.53 ± 2.38^b^	456.44 ± 23.20^bc^
	7653R	42.75 ± 1.50^a^	7.08 ± 0.59^a^	1.44 ± 0.14^a^	319.06 ± 24.25^a^	718.89 ± 64.51^a^
	CK	29.20 ± 1.33^c^	2.73 ± 0.17^c^	0.71 ± 0.06^c^	70.67 ± 26.10^c^	327.78 ± 46.27^c^
Minzi No.6	CCBAU2609	34.81 ± 1.17^c^	5.63 ± 0.10^b^	1.14 ± 0.08^b^	49.17 ± 31.87^b^	261.11 ± 40.44^b^
	M. h93	43.70 ± 1.26^b^	7.17 ± 0.74^b^	1.40 ± 0.16^b^	165.50 ± 109.59^ab^	696.67 ± 110.97^a^
	7653R	47.06 ± 0.83^a^	13.15 ± 1.26^a^	2.57 ± 0.29^a^	351.33 ± 105.19^a^	612.78 ± 102.89^a^
	CK	31.43 ± 0.46^d^	4.83 ± 0.31^b^	0.91 ± 0.10^b^	4.94 ± 1.62^b^	98.89 ± 38.22^b^
Minzi 8487711	CCBAU2609	53.55 ± 0.70^b^	6.68 ± 0.13^b^	1.25 ± 0.02^b^	5.67 ± 2.18^b^	106.11 ± 57.30^bc^
	M. h93	57.19 ± 1.49^ab^	7.88 ± 0.31^b^	1.43 ± 0.05^b^	29.17 ± 9.46^b^	317.78 ± 122.42^ab^
	7653R	67.08 ± 6.37^a^	10.89 ± 1.60^a^	1.96 ± 0.24^a^	312.22 ± 66.94^a^	551.11 ± 97.78^a^
	CK	51.04 ± 0.69^b^	6.22 ± 0.07^b^	1.26 ± 0.02^b^	0.56 ± 0.22^b^	32.22 ± 12.56^c^
Shishou variety	CCBAU2609	50.06 ± 0.22^c^	9.46 ± 0.16^b^	2.19 ± 0.10^a^	7.11 ± 3.62^b^	561.67 ± 67.50^b^
	M. h93	54.67 ± 0.37^b^	10.28 ± 0.10^b^	2.38 ± 0.32^a^	97.06 ± 35.13^b^	138.33 ± 52.24^a^
	7653R	58.74 ± 1.50^a^	15.37 ± 3.06^a^	3.03 ± 0.47^a^	323.00 ± 69.77^a^	709.44 ± 140.77^a^
	CK	39.11 ± 1.80^d^	5.38 ± 0.07^b^	1.13 ± 0.01^b^	0.89 ± 0.39^b^	178.33 ± 76.18^b^

**Data are means ± standard error around the mean. The columns were tested separately. The same letters indicate the lack of significant difference (P > 0.05), whereas different letters denote significant difference between corresponding values (P < 0.05).**

The further analysis of the nitrogenase activity index showed that the nitrogenase activity at full flowering stage of the different varieties of Chinese milk vetch inoculated with different strains of rhizobium was higher than that of CK (Table 6). The nitrogenase activity of Xinyang increased significantly by 3.38, 1.94, and 0.87 times after inoculation with 7653R, CCBAU 2609, and M. h 93, respectively. The nitrogenase activities of Minzi No.6, Minzi 8487711, and Shishou showed the highest increase after inoculation with 7653R and were 9.60, 6.21, and 15.18 higher than those of their respective CK plants. No significant difference in nitrogenase activity was observed when Minzi 6 was inoculated with CCBAU 2609 and M. h 93. Although the nitrogenase activity of Minzi 6 was significantly higher than that of CK, this increase was significantly lower than that of plants inoculated with 7653R. The nitrogenase activity of Minzi 84877711 had no significant change compared with that of CK plants after inoculation with CCBAU 2609 and M. h 93. The change trend of the nitrogenase activity of Shishou after inoculation with rhizobium was the same as that of Minzi No.6.

**TABLE 6:** Effect of different *Mesorhizobium huakuii* strains on the nitrogenase activity of Chinese milk vetch.

Strains	Nitrogenase activity (nmol C_2_H_4_⋅min^–1^⋅g^–1^)			
	Xinyang variety	Minzi No.6	Minzi 8487711	Shishou variety
CCBAU2609	448.64 ± 39.80^b^	201.05 ± 12.31^b^	50.38 ± 44.11^b^	406.02 ± 52.38^bc^
M. h93	284.45 ± 53.87^c^	252.87 ± 6.70^b^	175.37 ± 13.01^b^	727.98 ± 70.77^b^
7653R	734.21 ± 53.40^a^	793.05 ± 15.05^a^	409.12 ± 99.24^a^	1429.64 ± 236.13^a^
CK	152.46 ± 41.19^c^	74.80 ± 36.55^c^	56.76 ± 9.87^b^	88.34 ± 61.22^c^

**Data are means ± standard error around the mean. The columns were tested separately. The same letters indicate the lack of significant difference (P > 0.05), whereas different letters denote significant difference between corresponding values (P < 0.05).**

### Exclusivity Between Varieties of Chinese Milk Vetch and Strains of Rhizobium

**FIGURE 1:**
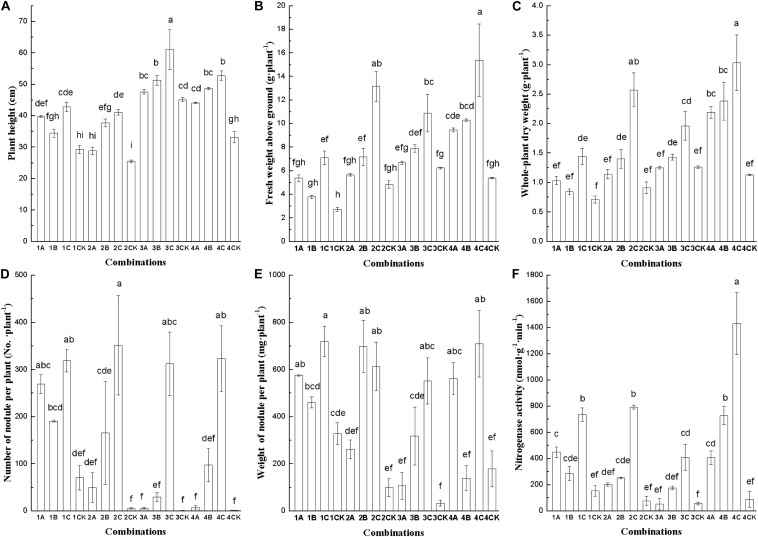
Plant height **(A)**, fresh weight above ground **(B)**, whole-plant dry weight **(C)**, nodule number per plant **(D)**, nodule weight per plant **(E)**, and nitrogenase activity **(F)** of Chinese milk vetch after inoculation with rhizobia. Numbers 1, 2, 3, and 4 indicate Xinyang variety, Minzi No.6, Minzi series 8487711, and Shishou variety, respectively. A, B, C, and CK represent strain CCBAU2609, M. h.93, 7653R, and control, respectively. Different lowercase letters indicate significant differences among treatments (*P* < 0.05).

Some differences in plant height, aboveground fresh weight, whole plant dry weight, nodule number and weight per plant, and nitrogenase activity were observed for different combinations of Chinese milk vetch varieties and rhizobium strains (Figure 1). The plant heights of 5 combinations, namely, Minzi 8487711–7653R, Minzi 8487711–M. h 93, Minzi 8487711–CCBAU 2609, Shishou–7653R, and Shishou–M. h 93, were higher than those of all other combinations (Figure 1A). The aboveground fresh weights of 4 combinations, including Shishou–7653R, Minzi 6–7653R, Minzi 8487711–Minzi 7653R, and Shishou–M. h 93, were higher than those of other combinations (Figure 1B). The whole-plant dry weight index of Shishou–7653R, Minzi No.6–7653R, Shishou–M. h 93, and Shishou–CCBAU 2609 were higher than those of all other combinations (Figure 1C). Six combinations, including Xinyang–CCBAU 2609, Xinyang–7653R, Minzi No.6–7653R, Minzi 8487711–7653R, Shishou–7653R, and Xinyang–M. h 93, had the highest number of nodules per plant (Figure 1D). Figure 1E shows that Xinyang–7653R, Xinyang–CCBAU 2609, Xinyang–M. h 93, Minzi No.6–M. h 93, Minzi 6–7653R, Minzi 8487711–7653R, Shishou–7653R, and Shishou–CCBAU 2609 had superior nodule weight indexes. Figure 1F depicts that 4 combinations, namely, Shishou–7653R, Shishou–M. h 93, Minzi No.6–7653R, and Xinyang–7653R, had higher nitrogenase activity than other combinations. On the basis of the above 6 indexes, Minzi No.6–7653R, Minzi 848711–7653R, Shishou–M. h 93, and Shishou–7653R were identified as combinations with strong exclusivity.

## Discussion

### Correlation Between Chinese Milk Vetch Varieties and Their Morphology, Yield, and Nitrogen Fixation Capability

Varieties of Chinese milk vetch, a non-main crop, are usually identified and approved by local authorities. In the 1970s and 1980s, the vigorous development of the chemical fertilizer industry nearly halted the research, promotion, and utilization of Chinese milk vetch. Most existing varieties are local varieties. Few national varieties have been tested across the whole country and approved by central authorities. Four local varieties of Chinese milk vetch were investigated in this study. The Shishou variety is from Shishou City, which is located in the middle and lower reaches of the Yangtze River. The Xinyang variety originates from Xinyang City, which is located at the Qinling Mountain and Huaihe River Line, 32°N. The two Minzi varieties are from Fujian Province. The mutual selection and adaption of each variety and origin factors, such as climate and soil, are combined with genetic characteristics. As a result, the morphology, grass yield, and nitrogen fixation capability of different varieties show some differences (Cho and Widholm, 2002a,b; Krasnyanskaya et al., 2002). These differences have been verified by the findings reported in this paper. The origin of Minzi varieties reaches 24°N, which is further south of the origin of the Shishou and Xinyang varieties. The effective cumulative temperatures during the growing season are high. Given that the general precipitation in China decreases from the southeast to the northwest, Fujian Province receives more precipitation than Shishou City, Hubei Province, and Xinyang City, Henan Province, the origins of the 2 other varieties. However, Shishou City and Xinyang City are, respectively, located on the edges of the Yangtze River and Huaihe River, where regional agricultural irrigation water can be effectively guaranteed. Therefore, these 2 varieties eventually became long-standing local species with large planting scales. According to the data from the National Experiment Network of Green Manure in 1980s, Chinese milk vetch can be divided into 4 types: earlier-flowering type, early flowering type, mid-flowering type, and late-flowering type. The Minzi No.6, Minzi 8487711, and Shishou varieties are mid-flowering types, whereas the Xinyang variety is an early flowering type. The measurement and analysis of growth processes and vegetative growth periods showed that plant height and grass yield peaked after budding and flowering. Therefore, the morphology, yield, and other indexes of the mid-flowering type were higher than those of the early flowering type as confirmed by the results of this work. Changes in climate, no matter the introduction from south to north or from north to south or mutual east–west introduction, result in changes in growth stage, morphology, yield, and even nitrogen fixation characteristics (Xu and Murooka, 1995; Long, 2001). For example, due to the high temperature in South China, the florescence of varieties introduced from the north to the south has been advanced. The growth stage of Ningbo Daqiao Chinese milk vetch, has reduced by 20 days after introduction into Fujian Province. The local variety from Zhejiang Province, which was introduced into the Yulin area of Guangxi Province 10 years ago, has shown reductions in plant height and yield (Chen and Shu, 1944). In addition to internal genetic factors, the differences in nitrogen fixation capability of varieties may also be related to the soil physicochemical properties of the long-term planting fields in the origin (Fauci and Dick, 1994; Melero et al., 2006; Jeong et al., 2019). With long-term evolution and adaptation, introduced varieties can exploit the advantage of high-efficiency nitrogen fixation when the soil conditions in the introduction area are similar to those in the origin. Under the condition that the Xinyang variety is planted in Nanjing without inoculation with exogenous rhizobium, the physical and chemical index of nodules are high likely because of similar soil conditions given that Nanjing and Xinyang are located in hilly and mountainous areas.

### Correlation Between Rhizobium and Chinese Milk Vetch Growth and Nodule Formation

Symbiotic nitrogen fixation between root systems and nodules plays a vital role in increasing the stem height, branch quantity, and yield of Chinese milk vetch, a leguminous plant (Faris et al., 1988; Bini et al., 2015; Liu et al., 2019). The rhizobium of Chinese milk vetch belongs to *Mesorhizobium huakuii* rhizobial species and not a permanent soil microflora (Chen et al., 1992). Exogenous rhizobium must be inoculated into fields that have not been planted with Chinese milk vetch or when the effect of indigenous rhizobium in the soil is not ideal (Lajudie et al., 1999). In this experiment, Chinese milk vetch showed differences in morphology, yield, and nitrogen fixation at different growth stages when inoculated with 3 strains of rhizobium (CCBAU 2609, M. h 93, and 7653R). These results revealed that different strains of rhizobia have different effects on plant growth and nitrogen fixation. Differences in morphology and yield were not significant when rhizobium was inoculated at the seedling stage. Differences were significant when rhizobium was inoculated at the full flowering stage. Most rhizobia affected nodule number and weight and nitrogenase activity and then further affected morphology and yield. Other studies have shown that the growth of Chinese milk vetch is positively correlated with nodules and soil nitrogen content. This correlation shows that fields with good population quality can promote nodule formation and efficient symbiotic nitrogen fixation. Nodules are visible 10 after inoculation (Zhu et al., 2002). During this period, the first true leaf unfolds, and white punctate nodules can be seen by the naked eye. However, the capability of nitrogen fixation does not appear until the early nodules have turned pink approximately 1 week later. Therefore, if the soil quality is poor, a small amount of nitrogen is often applied at the first leaf stage to start or accelerate symbiotic nitrogen fixation (Lerouge et al., 1990; Spaink and Sheeley, 1991). In addition, a certain correlation exists between the role of rhizobium in nodule formation and soil water. Although inoculating dry land with rhizobium also has significant effects, it is difficult because of the dry soil (Fatima et al., 2007). In production, the inoculation of rhizobium into dry land should be checked within 2 weeks after seedling emergence. If nodules are absent, supplemental inoculation should be done in time (Ligero et al., 2010). This work focused only on the effects of several strains of rhizobium on nodule number and weight and nitrogenase activity. The effects on nodule shape, formation time, color, and distribution; single or plural nodules; and the relevant environmental conditions affecting nodule formation by rhizobia were not investigated and thus need to be further verified in future research.

### Exclusivity Between Chinese Milk Vetch Varieties and Rhizobium Strains and Their High-Efficiency Combination

The rhizobium–legume mutualism system is unique. In this system, the legume provides mineral nutrition to the rhizobium, and the rhizobium provides nitrogen to the legume. Their mutualism is stable (Brett et al., 2010). Years of research have changed the previous view that it is the same rhizobium that infects all leguminous plants and causes nodule formation. Rhizobium strains have their own symbiotic exclusivity with specific leguminous plants, and different leguminous plants have their own corresponding rhizobium strains (Kulkarni and Nautiyal, 2000). This exclusivity is reflected not only by the different genera of leguminous plants, but also by the different varieties of the same species. However, some strains are known to be very promiscuous, such as *Sinorhizobium fredii* strain NGR_324_ which can nodulate legumes from different tribes and subfamilies in Fabaceae as well as the non-legume *Parasponia adansonii* (Perret et al., 2000). The relevant results of this work demonstrated that the nitrogenase activity of the 4 varieties inoculated with 7653R increased significantly, indicating that an efficient variety–strain combination could be established between the 4 varieties and 7653R. They also showed that 7653R was a broad-spectrum and efficient rhizobium that is suitable for the climate and soil conditions of the experimental site. In addition, CCBAU 2609 and Xinyang had higher exclusivity than Minzi No.6 and Shishou, and no exclusivity was found between CCBAU 2609 and Minzi 8487711. Although M. h 93 had a certain exclusivity with Shishou variety, this exclusivity was obviously weaker than the exclusivity shown by 7653R. Analysis also found that the good exclusivity between varieties and strains was best reflected by the index of nitrogenase activity and that exclusivity had a certain correlation with nodule number and weight, indicating that the strong nitrogen fixation capability of Chinese milk vetch depends on a certain number of nodules and large nodules (Pate, 1961; Chen et al., 1992). In this work, 4 varieties of Chinese milk vetch were inoculated with 3 strains of rhizobium. Several excellent combinations of symbiotic nitrogen fixation between Chinese milk vetch and rhizobium with good nodule growth and outstanding nitrogen fixation effect were selected. However, with the continuous change in soil and environmental conditions and the degradation of existing varieties and strain resources, we should, on the basis of the effective protection of existing strain resources, continue to screen excellent combinations of new nodule strains and additional varieties from the changing natural environment to improve the relevant indicators of nodulation and nitrogen fixation.

## Conclusion

(1) Under the condition of non-inoculation with exogenous rhizobium and in terms of the growth indexes at full flowering stage when vegetative growth and matter peaked, Xinyang and Minzi No.6 had obvious advantages in nodule weight and number per plant, and Minzi 8487711 and Shishou had certain advantages in plant height, aboveground fresh weight, and whole-plant dry weight. These results showed that Xinyang and Minzi No.6 had better adaptability to local indigenous rhizobia than other varieties, whereas the superiority of Minzi 8487711 and Shishou in morphology and yield was related to varietal characteristics.

(2) The comprehensive analysis of the plant height, yield, and nodule number and weight of different varieties inoculated with rhizobia at the seedling stage, branching stage, and full flowering stage revealed the following: First, exclusivity between Chinese milk vetch and rhizobium was weak at the seedling stage when exclusivity was only reflected by individual strains or individual indicators. Second, exclusivity at the branching stage was stable. Third, exclusivity was not attenuated at the full flowering stage.

(3) All the indexes of the 4 varieties inoculated with 7653R were significantly higher than those of their respective CK plants when yield peaked. The overall effect of the 4 varieties inoculated with 7653R was better than that of varieties inoculated with CCBAU 2609 and M. h 93, indicating that 7653R is a broad-spectrum and efficient rhizobium. In addition, the Xinyang variety inoculated with CCBAU 2609 had a good nitrogen fixation effect, and the nitrogenase activity of Shishou inoculated with M. h 93 was significantly higher than that of CK. This result indicated that directional inoculation can be done during production. The comprehensive analysis of morphology, yield, nodule characteristics, and nitrogenase activity showed that combinations, such as Minzi No.6–7653R, Minzi 848711–7653R, Shishou-M. h 93, and Shishou–7653R, have obvious advantages.

## Data Availability Statement

The original contributions presented in the study are included in the article/supplementary material, further inquiries can be directed to the corresponding author/s.

## Author Contributions

FL, YS, JL, and ZS conceived the ideas. FL, MY, and XL collected the data. FL and ZS analyzed the data and led the writing. HW and DY proofed the effectivity and rationality of the method proposed in this manuscript. All authors contributed critically to the ideas and drafts and gave final approval for publication.

## Conflict of Interest

The authors declare that the research was conducted in the absence of any commercial or financial relationships that could be construed as a potential conflict of interest.
